# Applying LFQRatio Normalization in Quantitative Proteomic Analysis of Microbial Co-culture Systems

**DOI:** 10.21769/BioProtoc.5294

**Published:** 2025-05-05

**Authors:** Mengxun Shi, Caroline A. Evans, Josie L. McQuillan, Josselin Noirel, Jagroop Pandhal

**Affiliations:** 1 School of Chemical, Materials and Biological Engineering, The University of Sheffield, Sheffield, UK; 2 GBCM Laboratory (EA7528), Conservatoire National des Arts et Métiers, HESAM Université, 2 rue Conté, Paris, France

**Keywords:** Proteomics, LFQRatio normalization, Label-free quantification, Quantitative proteomics, Microbial co-culture

## Abstract

Quantitative proteomic analysis plays a crucial role in understanding microbial co-culture systems. Traditional techniques, such as label-free quantification (LFQ) and label-based proteomics, provide valuable insights into the interactions and metabolic exchanges of microbial species. However, the complexity of microbial co-culture systems often leads to challenges in data normalization, especially when dealing with comparative LFQ data where ratios of different organisms can vary across experiments. This protocol describes the application of LFQRatio normalization, a novel normalization method designed to improve the reliability and accuracy of quantitative proteomics data obtained from microbial co-cultures. The method was developed following the analysis of factors that affect both the identification of proteins and the quantitative accuracy of co-culture proteomics. These include peptide physicochemical characteristics such as isoelectric point (pI), molecular weight (MW), hydrophobicity, dynamic range, and proteome size, as well as shared peptides between species. We then created a normalization method based on LFQ intensity values named LFQRatio normalization. This approach was demonstrated by analysis of a synthetic co-culture of two bacteria, *Synechococcus elongatus* cscB/SPS and *Azotobacter vinelandii* ΔnifL. Results showed enhanced accuracy of differentially expressed proteins, allowing for more reliable biological interpretation. This protocol provides a reliable and effective tool with wider application to analyze other co-culture systems to study microbial interactions.

Key features

• Assessment of factors affecting the quantitative accuracy of co-culture proteomics.

• Provides a LFQRatio normalization method for label-free quantification of microbial co-cultures.

• Recommendations for co-culture proteomics for mixed microbial populations.

## Background

Microbial co-cultures are valuable models for studying interactions between different microbial species, revealing insights into symbiotic relationships, competition, and metabolic cooperation [1–3]. Quantitative proteomics provides a critical tool that allows for the comprehensive analysis of protein dynamics in various biological systems, including microbial co-cultures.

Quantitative analysis in proteomics has largely shifted to label-free methods due to the rapidly increasing sensitivity of liquid chromatography (LC) and mass spectrometry hardware and the accuracy of proteomics data analysis tools [4]. Label-free quantification (LFQ) has advantages such as being cost-saving, requiring less stringent chemicals for extraction buffers, and presenting no limitations on the number of samples compared to label-based methods [5]. However, sample complexity and variations in protein abundance can make data interpretation difficult.

Many attempts have been made to deal with systematic biases among samples and to improve the accuracy of LFQ proteomics, including the creation of new algorithms [6–8]. However, there are challenges in applying these common workflows to synthetic co-cultures, i.e., when several strain types are cultivated together. This is particularly important when comparing conditions with highly variable cell-type ratios, as this will cause large differences in protein abundance between samples [9]. Therefore, a method for interpreting the contribution of two distinct protein groups in a sample in quantitative proteomics analyses remains to be established.

**Figure 1.**
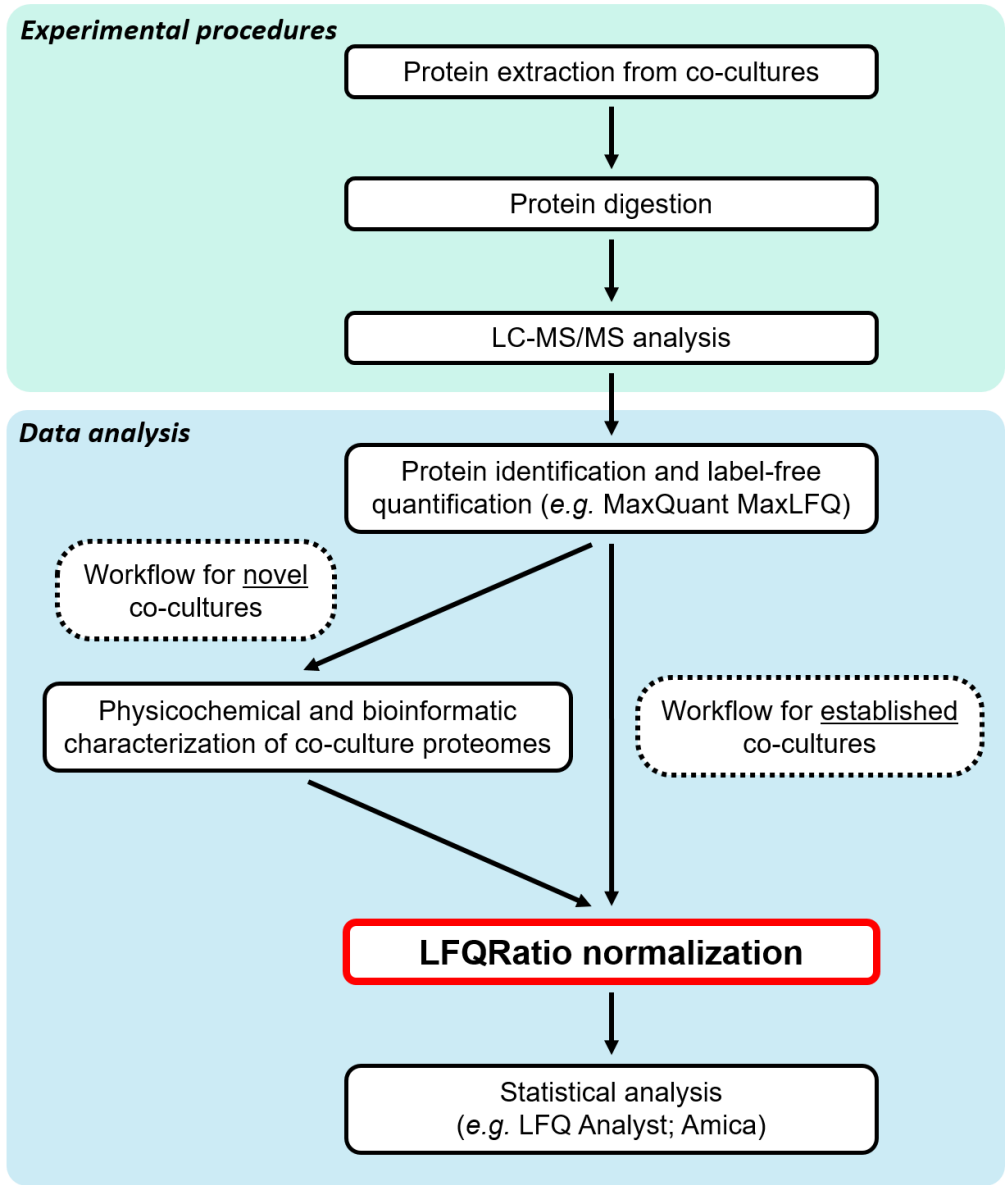
Label-free quantitative proteomics workflow with LFQRatio normalization for the analysis of co-cultures

**Figure 2.**
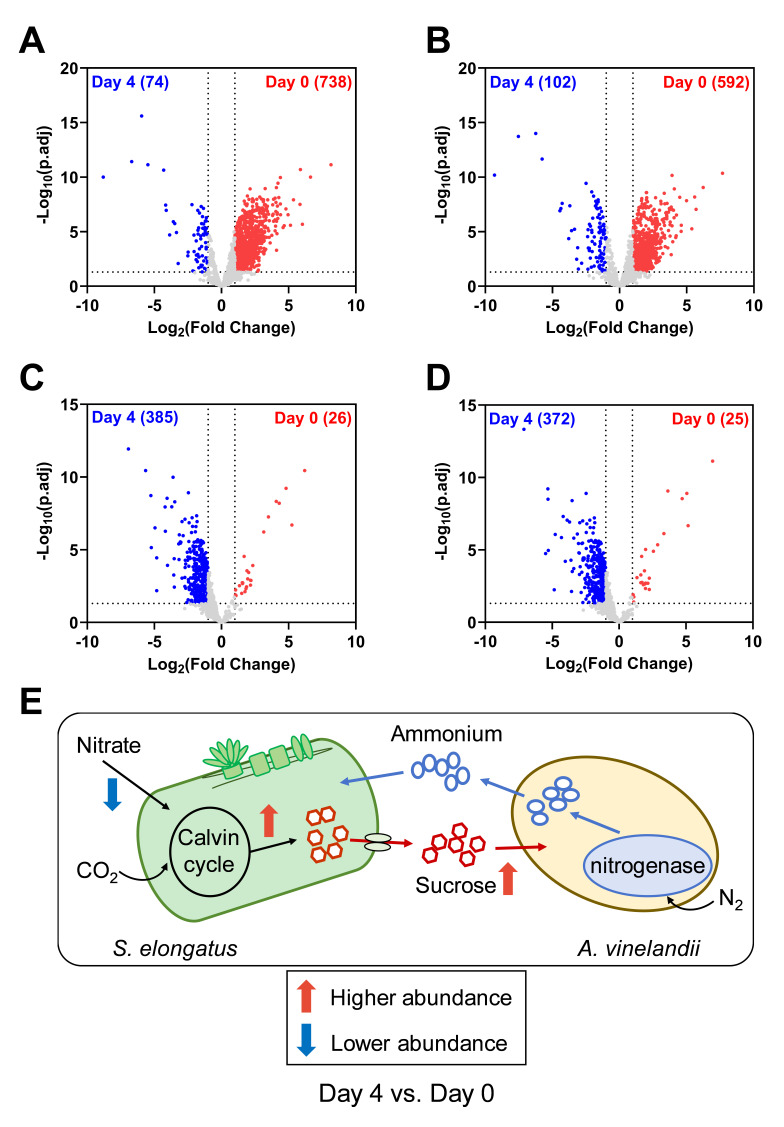
Example results obtained when including LFQRatio normalization in our label-free quantitative proteomics analysis pipeline for an *S. elongatus–A. vinelandii* co-culture. (A–D) Volcano plots showing differentially enriched proteins (DEPs) between Day 0 and Day 4 of an *S. elongatus–A. vinelandii* co-culture, with and without LFQRatio normalization. (A) DEPs of *S. elongatus* between Day 0 and Day 4 without LFQRatio normalization. (B) DEPs of *S. elongatus* between Day 0 and Day 4 with LFQRatio normalization. (C) DEPs of *A. vinelandii* between Day 0 and Day 4 without LFQRatio normalization. (D) DEPs of *A. vinelandii* between Day 0 and Day 4 with LFQRatio normalization. Each data point represents an individual protein. Proteins significantly enriched on Day 0 and Day 4 are shown in red and blue, respectively. Differential expression analysis was performed using LFQ Analyst using the following parameters: p-value cutoff = 0.05; Log_2_(fold change) cutoff = 1; imputation = Perseus style. (E) Schematic showing the changes in cross-feeding dynamics between Day 4 and Day 0 in an *S. elongatus–A. vinelandii* co-culture uncovered using LFQRatio normalization. On Day 4, nitrate uptake transporters in *S. elongatus* were less abundant, while proteins involved in sucrose production in *S. elongatus* and sucrose import in *A. vinelandii* were enriched, compared to Day 0. These biological conclusions were able to be drawn by applying LFQRatio normalization to the LFQ intensity values acquired using MaxQuant MaxLFQ; for more details, see Shi et al. [11].

To address these challenges, we started by analyzing the physicochemical properties and factors related to protein quantification between two co-cultured species. Then, we developed a new normalization method named LFQRatio, designed for the quantitative proteomic analysis of microbial co-culture systems (Figure 1). The LFQRatio normalization method is based on a more accurate LFQ intensity-based quantification approach [10, 11], which addresses the effects of changes in cell proportions between co-cultured microorganisms and facilitates comparisons of protein abundance under different conditions. This method enables more accurate identification of differentially expressed proteins, which is crucial for deciphering dynamics within microbial co-cultures (Figure 2).

## Materials and reagents

**Biological materials**

1. Bacterial monoculture 1: *Synechococcus elongatus* PCC 7942 cscB/SPS monoculture (from Abramson et al. [12]; kindly provided by Prof. Daniel Ducat). For growth monitoring information, see General notes

2. Bacterial monoculture 2: *Azotobacter vinelandii* DJ ΔnifL monoculture (from Ortiz-Marquez et al. [13]; kindly provided by Prof. Leonardo Curatti)

3. Bacterial co-culture (combination of bacterial monocultures 1 and 2): *Synechococcus elongatus* cscB/SPS and *Azotobacter vinelandii* ΔnifL co-culture

**Caution:** These strains are genetically modified and so need to be handled and disposed of according to the recommended safety guidelines of your institute.

**Reagents**

1. Sodium dodecyl sulfate (SDS) (Sigma-Aldrich, catalog number: 71725)

2. Phosphate-buffered saline (PBS) tablets (Millipore, catalog number: 6500-OP)

3. 100× Halt protease inhibitor cocktail (Thermo Scientific, catalog number: 78446)

4. 425–600 µm glass beads, acid-washed (Sigma-Aldrich, catalog number: G8772)

5. Urea, proteomics grade (Thermo Scientific, catalog number: 29700)

6. Trizma^®^ base (Sigma-Aldrich, catalog number: T1503)

7. Buffers for adjusting pH (e.g., NaOH/HCl) (Sigma-Aldrich, catalog number: 06203/258148)

8. DL-Dithiothreitol (DTT) (Promega, catalog number: V3151)

9. Water, HPLC grade (Fisher, catalog number: W/0106/PB17)

10. Iodoacetamide (IAA) (Sigma-Aldrich, catalog number: 1149)

11. Calcium chloride (CaCl_2_) (Sigma-Aldrich, catalog number: C3306)

12. Trifluoroacetic acid, HPLC grade (TFA) (Sigma-Aldrich, catalog number: 15608360 or similar)

13. Sequencing-grade modified trypsin (Promega, catalog number: V5111)

14. Formic acid, LC-MS grade (Thermo Scientific, catalog number: 85178)

15. Acetonitrile (ACN), HPLC grade (Fisher, catalog number: A/0627/17)

16. Methanol (MeOH), HPLC grade (Fisher, catalog number: M/4056/17)

**Solutions**

1. 1 M Tris-HCl (pH 8.5) (see Recipes)

2. 1 M DTT (see Recipes)

3. Lysis buffer (see Recipes)

4. Urea buffer (see Recipes)

5. 100 mM iodoacetamide (IAA) (see Recipes)

6. 1 mg/mL trypsin stock (see Recipes)

7. 100 mM CaCl_2 _(see Recipes)

8. 50 mM Tris-HCl (pH 8.5)/10 mM CaCl_2 _(see Recipes)

9. MS loading buffer (see Recipes)

10. 80% ACN (see Recipes)

11. MS mobile phase (see Recipes)

**Recipes**

*Note: Storage conditions are room temperature unless otherwise stated.*

**1. 1 M Tris-HCl (pH 8.5)**

Weigh 12.114 g of Tris and dissolve in 80 mL of HPLC-grade water. Lower the pH to 8.5 with HCl and top up to 100 mL with HPLC-grade water.

**2. 1 M DTT**

Dissolve 1.542 g of DTT in 10 mL of HPLC-grade water. Aliquot and store at -20 °C or use immediately.

*Note: DTT oxidizes in air, so the solution must be prepared fresh or frozen aliquots must be used immediately and then discarded.*

**3. Lysis buffer**

**Table:** 

Reagent	Final concentration	Quantity or Volume
SDS	2% (w/v)	400 mg
1 M Tris Tris-HCl (pH 8.5)	40 mM	0.8 mL
1 M DTT	60 mM	1.2 mL
HPLC-grade water		Up to 20 mL

**4. Urea buffer**

**Table:** 

Reagent	Final concentration	Quantity or Volume
Urea	8 M	240 mg
1 M Tris-HCl (pH 8.5)	100 mM	50 µL
1 M DTT	5 mM	2.5 µL
HPLC-grade water		Up to 500 µL

*Note: We recommend using 1.5 mL LoBind Eppendorf tubes to prepare the solution. Always make up fresh urea solution. When in solution, urea degrades within a few hours into a compound that can alter the composition of your protein sample.*

**5. 100 mM iodoacetamide (IAA)**

Dissolve 0.0185 g of iodoacetamide in 1 mL of HPLC-grade water.

*Note: Always prepare fresh as IAA is light-sensitive. Wrap in foil once prepared.*

**6. 1 mg/mL trypsin stock**

Add 20 µL of resuspension buffer (provided by the trypsin manufacturer) to one vial of lyophilized sequencing-grade modified trypsin (20 µg) to generate the trypsin stock. Store at -80 °C for up to 1 month.

**7. 100 mM CaCl_2_**

Dissolve 1.47 g of CaCl_2_ in 100 mL of HPLC-grade water.

**8. 50 mM Tris-HCl (pH 8.5)/10 mM CaCl_2_**

**Table:** 

Reagent	Final concentration	Quantity or Volume
1 M Tris-HCl (pH 8.5)	50 mM	1 mL
100 mM CaCl_2_	10 mM	2 mL
HPLC-grade water		17 mL

**9. MS loading buffer**

**Table:** 

Reagent	Final concentration	Quantity or Volume
ACN	3% (v/v)	30 µL
TFA	0.1% (v/v)	1 µL
HPLC-grade water		969 µL

**Caution:** Prepare in a fume cabinet.

**10. 80% ACN**

**Table:** 

Reagent	Final concentration	Quantity or Volume
ACN	80% (v/v)	800 mL
HPLC-grade water		200 mL

**Caution:** Prepare in a fume cabinet.

**11. MS mobile phase**

**Solvent A**

**Table:** 

Reagent	Final concentration	Quantity or Volume
Formic acid	0.1% (v/v)	1 mL
HPLC-grade water		999 mL

**Solvent B**

**Table:** 

Reagent	Final concentration	Quantity or Volume
Formic acid	0.1% (v/v)	1 mL
80% ACN		999 mL

**Caution:** Prepare in a fume cabinet.

**Laboratory supplies**

1. 1,000 µL pipette tips

2. 200 µL pipette tips

3. 10 µL pipette tips

4. 50 mL sterile conical tubes (Falcon, catalog number: 352070)

5. 1.5 mL protein LoBind tubes (Eppendorf, catalog number: 0030108116)

6. 2D Clean-Up kit (Cytiva, formerly GE Healthcare Life Sciences, catalog number: 80-6484-51)

7. Pierce^TM^ C18 spin columns (Thermo Scientific, catalog number: 89870)

8. 0.3 µL PP snap ring HPLC vials (VWR, catalog number: 548-0120A)

9. ND11 open-top PE HPLC vial snap caps (VWR, catalog number: 548-3203A)

## Equipment

1. Pipettes (p1000, p200, p10, p2.5) (Eppendorf)

2. Spectrophotometer (Jenway, model: 6305)

3. Temperature-controlled centrifuge (e.g., Thermo Scientific, model: SL 1ER)

4. Vortex with attachment for multiple microfuge tubes (Scientific Industries, model: Vortex-Genie 2)

5. Heated water bath (e.g., Grant, model: JB Nova)

6. Ultrasonic water bath (Fisherbrand, model: FB15051)

7. Nanodrop spectrophotometer (Thermo Scientific, model: Nanodrop 2000)

8. Incubator capable of maintaining 30–37 °C (e.g., Thermo Scientific, model: MaxQ 6000)

9. Vacuum concentrator (Eppendorf, model: Concentrator Plus)

10. Nano-flow liquid chromatography (Thermo Fisher Scientific, model: U3000 RSLCnano)

11. Hybrid quadrupole-orbitrap mass spectrometer (Thermo Fisher Scientific, model: Q Exactive HF)

12. Easy-Spray C18 column, 75 µm × 50 cm, Pepmap RSLC, 2 µm, 100 Å (Thermo Scientific, catalog number: E5903)

## Software and datasets

1. Uniprot (https://www.uniprot.org/, 02/03/2020)

2. MaxQuant (2.0.3.0)

3. RStudio (4.2.0)

4. LFQ-Analyst (https://analyst-suite.monash-proteomics.cloud.edu.au/apps/lfq-analyst/, 08/09/2023)

5. All code has been deposited to GitHub: https://github.com/Mengxun-Shi/bio-protocol (01/13/2025)

## Procedure

**A. Protein extraction**

1. Grow cell cultures to a growth phase appropriate for your experiment and measure the optical density at the wavelength suitable for your culture. Centrifuge 20 mL of cells at 4,000× *g* for 10 min at 4 °C in 50 mL centrifuge tubes.

*Note: We grew our co-cultures in 500 mL flasks over an 8-day period, sampling on Days 0, 4, and 8 [11] (see General note 1). However, we encourage the reader to select their harvest points based on their own biological questions and comparisons.*

2. Wash the pellets by resuspending in 10 mL of PBS buffer and repeating the centrifugation. Pellets can be stored at -20 °C for up to 1 month before processing.

3. Resuspend the pellet in 500 µL of lysis buffer per 1 OD unit (e.g., if OD = 2, add 1 mL of lysis buffer) and add the appropriate amount of 100× Halt protease inhibitor cocktail (e.g., if the total volume of the pellet + lysis buffer is ~700 µL, add 7 µL of protease inhibitor).

4. Freeze the cells at -80 °C for >16 h and quickly thaw them in a water bath at 37 °C for 3–5 min to allow partial cell breakage. Immediately transfer to ice after thawing.

*Note: The frozen pellets can remain at -80 °C in lysis buffer for ~1 month.*

5. Label and pre-cool 1.5 mL LoBind microcentrifuge tubes on ice. Transfer the lysed cell sample to the LoBind tubes. For the remainder of the procedure, keep the samples on ice.

6. Add approximately 500 µL of 425–600 µm acid-washed glass beads to the samples, leaving 2–3 mm of cell suspension above the level of settled beads.

7. Break the cells by vigorous vortex mixing of the tube 20 times in cycles of vortexing for 30 s and cooling on ice for 30 s using a vortex with a multi-tube attachment.

8. Incubate samples at 95 °C for 5 min using a dry heat block.

9. Collect the supernatant in a fresh 1.5 mL LoBind tube following centrifugation at 15,000× *g* for 10 min at 4 °C. The crude protein sample can be stored at -20 °C for ~1 month.

*Note: At this point, it would be a good idea to quantify your crude proteins. Our crude protein concentration was typically 2–3 mg/mL. This can be done using, for example, a detergent-compatible Bradford reagent (Pierce, catalog number: 23246). It is also advisable to run the protein samples on an SDS-PAGE gel.*

10. Purify the crude samples using a 2D Clean-Up kit following the manufacturer’s instructions to remove interfering substances and, if required, to concentrate the proteins. The resuspension buffer will be the urea buffer (see Section B).

**Caution:** Note the hazard statements in the 2D Clean-Up kit for the precipitant and wash buffer, which include “causes severe burns and eye damage” and “suspected to cause cancer.”

*Note: At the wash step, the protein pellet can be stored in wash buffer and wash reagent for up to 1 week at -20 °C. However, once the pellet has been dried for <5 min, it is advisable to immediately proceed to the digestion steps and resuspend the pellet in urea buffer.*

**B. Protein digestion**

1. Dissolve protein pellet in 50 µL of urea buffer. Sonicate the sample in an ultrasonic water bath to solubilize the proteins; the suspension should be transparent.

*Note: If the sonication step takes longer than 10 min, cool the samples on ice for 5 min and continue the sonication until the samples are transparent.*

2. Take 1 µL of resuspended protein pellet and dilute 1:10 by adding 9 µL of H_2_O. Estimate protein concentration using the Nanodrop: Use 1 µL of 1:10-diluted urea buffer as a blank. Add 1–2 µL diluted protein sample to the Nanodrop to estimate protein concentration at 280 nm.

3. Reduction step: Transfer 50 µg of protein to a fresh 1.5 mL LoBind tube. Dilute to 10 µL with urea buffer. Incubate at 37 °C in a water bath for 30 min.

*Note: DTT, the reducing agent, is included in the urea buffer.*

4. Alkylation step: Add 1.5 µL of 100 mM IAA to the protein solution and incubate in the dark at room temperature for 30 min.

5. Digestion step: Add 1 µL of 1 mg/mL sequencing-grade modified trypsin stock to the protein sample and then add 67.5 µL of 50 mM Tris-HCl (pH 8.5)/10 mM CaCl_2_ to a total volume of 80 µL. Digest overnight at 37 °C.

*Note: The protease:protein ratio recommended by the trypsin manufacturer is 1:100 to 1:20 (w/w); here, we used 1:50.*

6. Terminate the digestion: Add 0.8 µL of formic acid to a final concentration of 1% (recommend 0.1–1% v/v). Once added, it can be stored at -20 °C for up to 48 h.

7. Use C18 Spin columns to purify the digested peptides and remove contaminants following the manufacturer’s instructions. Purified samples should be dried using a vacuum concentrator and stored at -20 °C (days/weeks) or -80 °C (months) until mass spectrometry analysis.

*Note: The binding capacity of the C18 Spin columns is 30 µg. By overloading the column with 50 µg of peptides, 30 µg will reliably be captured and eluted without peptide loss.*

**C. Mass spectrometry**

1. Dissolve dried peptide pellets in 30 µL of loading buffer for a peptide concentration of 1 µg/µL. Sonicate samples in a water bath for 3 min at room temperature to suspend the samples.

2. Centrifuge the sample at 15,000× *g* for 2 min. Take 5 µL from the top of the centrifuged sample and transfer to an HPLC vial. Top up with 15 µL of loading buffer (20 µL total volume; 250 ng/µL peptide concentration), ensuring not to introduce bubbles to the vial.

3. Inject 500 ng of purified peptides (2 µL) into the LC–MS system with Easy-Spray C18 column. Perform LC–MS/MS by nanoflow liquid chromatography coupled to a hybrid quadrupole-orbitrap mass spectrometer or equivalent LC–MS/MS equipment. Here, peptides were separated by reverse-phase high-performance liquid chromatography using an Easy-Spray C18 column (75 µm × 50 cm) at a flow rate of 300 nL/min and column temperature of 40 °C. Two mobile phases were used: mobile phase A was composed of water with 0.1% formic acid v/v, and mobile phase B was composed of 80% acetonitrile in water, 0.1% v/v formic acid. Both were mixed over time for gradient elution. A 2-step gradient was employed: from 3% B to 10% B over 5 min, then from 10% B to 50% B (0.1% formic acid in 80% acetonitrile) to 75 min.

4. The mass spectrometer was programmed for data-dependent acquisition with 10 product ion scans (resolution 30,000, automatic gain control 1 × 10^5^, maximum injection time 60 ms, isolation window 2.0 m/z, fixed first mass 100 m/z, normalized collision energy 28, and intensity threshold 3.3 × 10^4^) per full MS scan (resolution 120,000, automatic gain control 1 × 10^6^, maximum injection time 60 ms, scan range 375–1,500 m/z) with a 35 s dynamic exclusion time. Make sure to include both biological (≥3) and technical (>2) replicates and ensure the appropriate control samples are included for meaningful sample comparison(s) to ensure the biological question is addressed.

## Data analysis

**A. Protein identification and quantification**

1. Create a reference database (FASTA format) using all protein sequences of both strains; in this case, *S. elongatus* PCC 7942 and *A. vinelandii* DJ appended with the amino acids sequences of sucrose permease (CscB) from *Escherichia coli* and sucrose phosphate synthase (SPS) from *Synechocystis* sp. PCC6803 from Uniprot (Dataset S3).

*Note: This can be done with a simple notepad program, saving the file name with the appendage .fasta.*

2. Load the raw MS data files and database to MaxQuant (2.0.3.0) [7].

3. Select modifications: *Oxidation (M*) and *Acetyl (Protein N-term*) for variable modification, and *Carbamidomethyl (C)* for fixed modification.

4. Select *Trypsin/P* for enzymatic digestion.

5. Filter peptide-spectrum matches and protein identifications using a target-decoy approach at a false discovery rate (FDR) of 1%.

6. Select *LFQ* and intensity-based absolute quantification (*iBAQ*) options for quantitative analysis. Other parameters are shown in the supplementary files (Table S1).

7. If your synthetic co-culture is a novel combination of two bacteria, and this is the first proteomics experiment conducted for this consortium, continue to Section B. Otherwise, proceed to Section C.

**B. Assessment of factors relating to protein identification for first-time proteomic analyses of new synthetic co-cultures**

The physicochemical characteristics of proteins, such as the range of pI [14], MW [15], and hydrophobicity [16], as well as the dynamic range distribution of protein abundances within the proteome [17], can affect factors such as protein extraction efficiency and ionization efficiency in the mass spec, and hence how many peptides are detected. Similarly, a much larger proteome or highly abundant protein could mean a bias toward identifying peptides from a specific organism [18]. Finally, shared peptides between the strains will conflict with quantitative data and so should be taken into account [19]. We therefore advise researchers to identify variations in these factors between co-culture species, which could affect quantitative accuracy and therefore the biological interpretation of LFQ proteomics data.

1. Preliminary physicochemical characteristics analysis

a. Assess the theoretical pI ranges of the proteins in *S. elongatus* and *A. vinelandii* using R script (File S1).

b. Assess the MW of the proteins in *S. elongatus* and *A. vinelandii* using R script (File S2).

c. Assess the hydrophobicity of proteins in the *S. elongatus* and *A. vinelandii* databases via the grand average of hydropathy (GRAVY) scores using R script (File S3).

d. Dynamic range: Quantify the abundance of detected *S. elongatus* and *A. vinelandii* proteins by absolute quantification (iBAQ). Use the values from MaxQuant outputs–ProteinGroups.txt file, calculating log10 iBAQ intensity to show the dynamic range of both strains. Plot log10 iBAQ intensity against protein number.

*Note: The dynamic range is the range of MS1 peak intensities over which peptides can be detected. A wide dynamic range or a protein that is extremely abundant in one strain may affect the quantification of the co-culture proteome. Therefore, it is critical to examine the dynamic range of proteins for each strain in the microbial community before further analysis [11].*

2. Proteome size analysis

a. Run MaxQuant multiple times against *S. elongatus* and *A. vinelandii* individual and merged databases. For example, we run MaxQuant four times: i) *S. elongatus* monoculture against the *S. elongatus* database (Dataset S1); ii) *S. elongatus* monoculture against the merged database (Dataset S3); iii) *A. vinelandii* monoculture against the *A. vinelandii* database (Dataset S2); and iv) *A. vinelandii* monoculture against the merged database (Dataset S3).

b. Compare the identified protein numbers of *S. elongatus* and *A. vinelandii* taken from proteinGroups.txt files of each run. Filter for proteins with two or more unique peptides.

*Note: We expect the same strain to have the same/similar number of proteins identified in both individual and combined database runs, which means that database size does not affect protein identification in this case. However, this should be considered in co-culture proteomics workflows, particularly where proteome sizes of co-culture members vary more widely.*

3. Shared peptides analysis

a. Calculate theoretical shared peptides using R script (File S4) of tryptic peptides of the specified size (8–25 amino acids).

b. Analyze measured shared peptides by comparison of the resulting peptide sequences of each strain from MaxQuant outputs–Peptides.txt file.

**C. LFQRatio normalization and proteomic analysis**

1. Open the ProteinGroup.txt file output from MaxQuant and select identified proteins with two or more peptides.

2. Normalize LFQ intensity data using the LFQRatio normalization method as shown in the following equation, i.e., for each individual protein detected, its LFQ intensity is divided by the sum of all protein LFQ intensities for its respective strain:

$$Normalized\;LFQ\;intensity=\frac{LFQ\;intensity\;of\;one\;protein}{total\;LFQ\;intensities\;of\;the\;strain}$$

3. Use the normalized proteomics data to analyze differentially expressed proteins using an LFQ proteomics analysis platform such as LFQ-Analyst [20]. The following steps will be for using LFQ-Analyst; however, other platforms can be used (see General notes).

4. Load two input files: the LFQRatio-normalized proteinGroups.txt file and an experimental design file (an exemplary experimental design file can be downloaded from the LFQ-Analyst website).

5. In the *Advanced Options*, the adjusted p-value cutoff and Log2 fold change cutoff can be selected; we applied the default options of 0.05 and 1, respectively.

6. The default imputation type (Perseus type) can be applied; however, this is not necessarily recommended (see General notes and troubleshooting)

7. The type of FDR correction can be selected; we applied Benjamini–Hochberg for our experiment.

8. A list of differentially expressed proteins for each pair-wise comparison will be generated, as well as quality control plots, volcano plots, and expression heatmaps. Example volcano plots showing differential expression data generated with and without application of LFQRatio are shown in Figure 2.

9. Other parameters are shown in Table 1.

**Table 1.** Parameter settings of LFQ-Analyst used for differentially expressed proteins analysis

Parameters	Settings
Adjusted p-value cutoff	0.05
Log2 fold change cutoff	1
Imputation type	Perseus type* (see General notes)

## Validation of protocol

This protocol or parts of it has been used and validated in the following research article(s):

• Shi et al. [11] LFQRatio: A normalization method to decipher quantitative proteome changes in microbial coculture systems. Journal of Proteome Research (Figure 9).

• Kratzl et al. [21] *Pseudomonas putida* as saviour for troubled *Synechococcus elongatus* in a synthetic co-culture–interaction studies based on a multi-OMICs approach. Communications Biology (Figure 4).

## General notes and troubleshooting

1. Growth details for *S. elongatus* and *A. vinelandii:* In our model system, we performed a time-course experiment to examine the growth and physiology of our *S. elongatus* and *A. vinelandii* co-culture over 8 days, harvesting samples for proteomics analysis on Days 0, 4, and 8. We monitored the growth both by optical density (OD) at 750 nm, which did not distinguish between strains but showed overall growth of the system, and by i) flow cytometry for *S. elongatus* and ii) calculating colony forming units for *A. vinelandii*. For more information on media, starting cell ratios, growth monitoring, and maintenance of these strains, we direct the reader to our paper [11].

2. Alternative in-browser programs for analyzing differentially expressed proteins: LFQ-Analyst was suggested here as an accessible, in-browser option for performing differential expression analysis of LFQ proteomics data [20]. Since performing our initial analysis [11], a developer version of LFQ-Analyst has been released (https://analyst-suites.org/apps/lfq-analyst-dev/), which allows for more control over parameter settings for differential expression analysis, as well as presence/absence analysis. Another powerful in-browser tool that we recommend is amica, which provides the user with an in-depth quality control analysis and parameter selection [22].

3. Dealing with missing values: Missing values in LFQ proteomics datasets are to be expected [23]. They can be broadly categorized as either “missing not at random” (MNAR), where the protein is either not expressed or has an abundance lower than the mass spectrometer’s limit of detection (i.e., biological reasons), or “missing at random” (MAR), where the protein is not identified for reasons related to the protocol and instrumentation (for more detail, see Jin et al. [24]). So as not to lose valuable data, we can apply various imputation algorithms to *fill in* these missing values.

We observed that missing values were particularly prevalent in mixed co-culture samples compared to their mono-culture counterparts, and the extent of this will likely differ between co-culture types. In our example application of LFQRatio, we applied the default imputation settings (Perseus type) for our DEP analysis [11]; we also applied this same imputation method in another study [21] in which we achieved a good correlation of the proteomics data with transcriptomics data, suggesting that this imputation method was acceptable. However, we advise researchers to consider which imputation method is most appropriate for their own dataset; for more information and guidance, we refer the reader to this paper: https://www.nature.com/articles/s41598-021-81279-4.

4. LFQ-Analyst details: LFQ-Analyst analyzes differentially expressed proteins according to the following steps: Filter out contaminant proteins, reverse sequences, and proteins identified “only by site;” remove proteins that have been only identified by a single peptide and proteins not identified/quantified consistently in the same condition; transform the LFQ data to log2 scale; group samples by conditions and missing values imputed using the selected imputation type [here, we used Perseus-type imputation, a MNAR method that takes random draws from a left-shifted Gaussian distribution of 1.8 StDev (standard deviation) apart with a width of 0.3]; use protein-wise linear models combined with empirical Bayes statistics for the differential expression analyses; and use the limma package from R Bioconductor to generate a list of differentially expressed proteins for each pair-wise comparison [20].

## Supplementary information

The following supporting information can be downloaded here:

1. Table S1. Parameter settings of MaxQuant software used for protein identification and quantification.

2. File S1. R script for analyzing the pI of *S. elongatus* or *A. vinelandii* proteome.

3. File S2. R script for analyzing the MW of *S. elongatus* or *A. vinelandii* proteome.

4. File S3. R script for analyzing the hydrophobicity of *S. elongatus* or *A. vinelandii* proteome.

5. File S4. R script for analyzing shared peptides between *S. elongatus* and *A. vinelandii.*

*Note: These scripts can be used for other organisms by changing the “sequence.fasta” file to the corresponding FASTA files.*

6. Dataset S1. Protein sequences of *S. elongatus* cscB/SPS.

7. Dataset S2. Protein sequences of *A. vinelandii* ΔnifL.

8. Dataset S3. Protein sequences of *S. elongatus* cscB/SPS and *A. vinelandii* ΔnifL co-culture (merged database).
